# HAART rollout in the new fiscal and economic environment

**DOI:** 10.7448/IAS.15.6.18081

**Published:** 2012-11-11

**Authors:** J Moatti

**Affiliations:** Aix-Marseille University, Marseille, France

## Abstract

The trend towards universal access for HIV prevention and treatment that was initiated at the beginning of the 21st century (international donor funding has been multiplied by 3 to reach 27 billion US$ in 2010) has been threatened by the 2008–9 economic crisis which currently translates in a fiscal crisis for most developed countries (including the US, France and the UK – the main donors for HIV/AIDS). Other advances such as the drastic drop in ARV drug prices are also threatened (generic first line drugs close to marginal cost, insufficient drop in second line drug prices, etc.). The presentation will discuss the negative consequences of slowing down and delaying universal access on macro-economic growth in the most affected countries and suggest alternative sources of funding such as the financial transaction tax recently introduced by the French Parliament.

